# Monogenic Diabetes Secondary to Congenital Lipodystrophy in a 14−year−old Yemeni Girl

**DOI:** 10.4274/jcrpe.v2i4.176

**Published:** 2010-11-09

**Authors:** Todd Roth, Sri Nair, Anıl Kumar

**Affiliations:** 1 VCUHS, Pediatrics, Richmond VA, USA; +91 804 201 83 32tsroth2117@gmail.comVCUHS, Pediatrics, Richmond VA, USA

**Keywords:** Congenital generalized lipodystrophy, Berardinelli−Seip congenital lipodystrophy type 2, insulin resistance, monogenic diabetes, leptin

## Abstract

A 14−year−old female from Yemen presented with intense abdominal pain and headache. She was born at term to distant cousins, developmentally delayed and significantly dysmorphic. Four years ago, she was diagnosed with diabetes mellitus and undiagnosed hepatic, cardiac, genetic, neurologic, endocrine, musculoskeletal, and gastrointestinal disorders. No therapy was prescribed. Admission laboratory data showed blood glucose = 391 mg/dl, hemoglobin A1c= 12.2%, C−peptide = 3.5ng/ml, insulin = 6.8 uIU/ml, triglyceride =385mg/dl, and serum leptin <0.5ng/ml, (1.1−27.5). Chromosome analysis (46, XX) was normal and serology for Glutamic acid Decarboxylase (GAD), hepatitis and HIV were negative. Clinical examination and laboratory data suggested congenital generalized lipodystrophy (CGL, type BSCL−2). This case illustrates that CGL should be in the differential diagnosis for non−obese patients with diabetes and insulin resistance.

**Conflict of interest:**None declared.

## INTRODUCTION

Congenital generalized lipodystrophy (CGL), also known as Berardinelli−Seip congenital lipodystrophy (BSCL), was first described by this author in 1954 (1). It is a rare genetic disorder that has only been reported in 300 patients (2). Its prevalence in the United States is 1 per 12 million (3). Two subtypes of BSCL, designated as type 1 and type 2, have been defined. Magré et al (4) identified a locus linked to this disease on chromosome 11q13, and labeled it BSCL2, which corresponds to type 2 BSCL. This type is characterized by a generalized lipoatrophy, not restricted to the subcutaneous area (5), that is associated with significant insulin resistance and its sequelae, namely, diabetes, hyperinsulinemia, dyslipidemia, hepatic steatosis, acanthosis nigricans, polycystic ovaries, and hypertension (6, 7, 8). There may be additional pathology such as cardiomyopathy, developmental delay, bone cysts, nephromegaly and nephropathy (9, 10, 11, 12, 13, 14). The aim of this paper was to present this illustrative BSCL patient and underline that CGL should be considered in the differential diagnosis of non−obese patients with diabetes and insulin resistance.

## CASE REPORT

A 14−year−old girl, offspring of a family who recently moved from Yemen to the United States, presented with symptoms of abdominal pain, polyuria and polydipsia, which had started a few weeks ago. The severity of the abdominal pain, described as dull, diffuse, and present for a long time, had increased in the past 2 days. The patient was reported to have had a protuberant abdomen since birth, which had shown no change in the recent past. She had frequent voidings that worsened over the last few weeks. She had a very good appetite. Ingestion of fluids was reported as adequate. The patient was born full term and had had no perinatal problems, but was developmentally delayed (talked and walked at age 4 years). At 8 years of age, she was diagnosed to have diabetes mellitus (DM) in Jordan by routine blood tests that showed a high blood sugar. Hypertension was diagnosed a few years later. The parents were told that the prognosis was poor. No treatment was offered for DM or hypertension. Breast development and pubic hair were noticed at age 9 years and axillary hair and body odor − at age 11 years. She had increased facial and body hair, but the age at which this was noticed was not clear. The patient had not yet started her menstrual periods. She had a history of poor dental hygiene and poor vision. The family history revealed a consanguineous relationship (distant cousins) between the father and mother who were otherwise healthy. The patient has three sisters and one brother who are all reported to have no medical problems. There is no family history of congenital or metabolic diseases. A review of the symptomatology is listed in Table 1, and the findings on physical examination are given in Table 2. Vital signs at presentation were: pulse: 98/min, blood pressure: 144/84 mmHg, respiration: 20 /min, afebrile. She weighed 40.3 kg (5^th^ percentile for age, −1.7 SD) and her height was 144 cm (under the 3rd percentile, −2.25 SDS), her body mass index(BMI) was 19.4 kg /m^2^ (0.00 SD). The pertinent features were: absence of subcutaneous fat in the extremities, face, trunk and hands; prominent muscles; looking much older than her stated age; dysmorphic features; developmental delay; hepatosplenomegaly; poor vision; enophthalmus; and arthropathy. There was no evidence of retinopathy. Laboratory data are listed in Table 3. Her serum glucose was 393 mg/dL and there was no metabolic acidosis. Given the history of fever and hepatosplenomegaly, screening for human immunodeficiency virus, Hepatitis A, B, C, and tuberculin test were done, and were all negative. Imaging studies demonstrated striking organomegaly involving the liver, spleen, and kidneys. Stool impaction was evident on X−ray. Ultrasound of the abdomen showed hepatomegaly, enlargement of the main portal vein, numerous splenic varices, and splenomegaly indicating a mild degree of portal hypertension. Radiograms showed mottled bones. Echocardiogram revealed aortic insufficiency and left ventricular hypertrophy. Chromosomal analysis specifically for mutation of 11q13 and 9q34 is not yet completed.

The clinical features and laboratory data of this patient suggested a diagnosis of congenital lipodystrophy, and more specifically of BSCL2. She was started on an insulin regimen of 1 unit/kg/day and increased up to 6 units/kg/day (300 units daily). Her present insulin regimen includes glargine (100 units) at bedtime and aspart (40−60 units) before each meal. Given the risks of liver toxicity associated with metformin, metformin was started at 250 mg once a day and gradually increased to 500 mg twice a day. Liver function tests were monitored every 6 weeks. Even with combined insulin and metformin therapy, her blood sugar has remained in the 200−500mg/dL range. Her hemoglobin A1c (HbA1c), which was 12.2% at diagnosis, had improved to 10.6% over a 6−month period. She is encouraged to do more physical activity. She was also started on ergocalciferol and calcium for vitamin D deficiency. The carious teeth were extracted. For persistent proteinuria and nephromegaly, a kidney biopsy was done that showed IgA nephropathy. She was placed on angiotensin−converting enzyme inhibitor for persistent proteinuria and hypertension. Her blood pressure has normalized on antihypertensive medication. Portal hypertension has been monitored closely as these patients are at risk for esophageal varices.

**Table 1:**
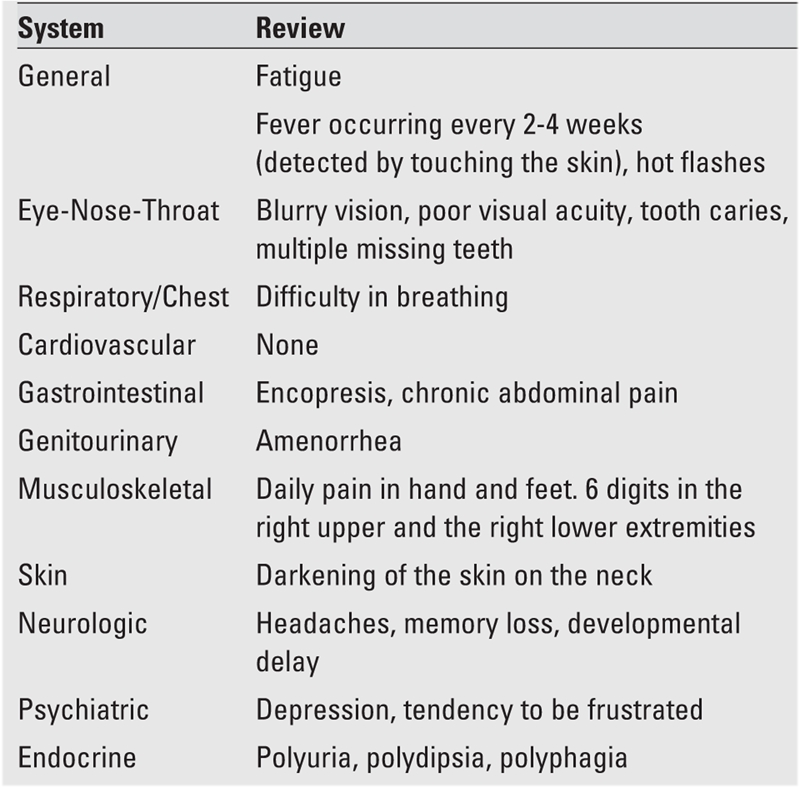
Reported symptoms in the history of the patient

**Table 2:**
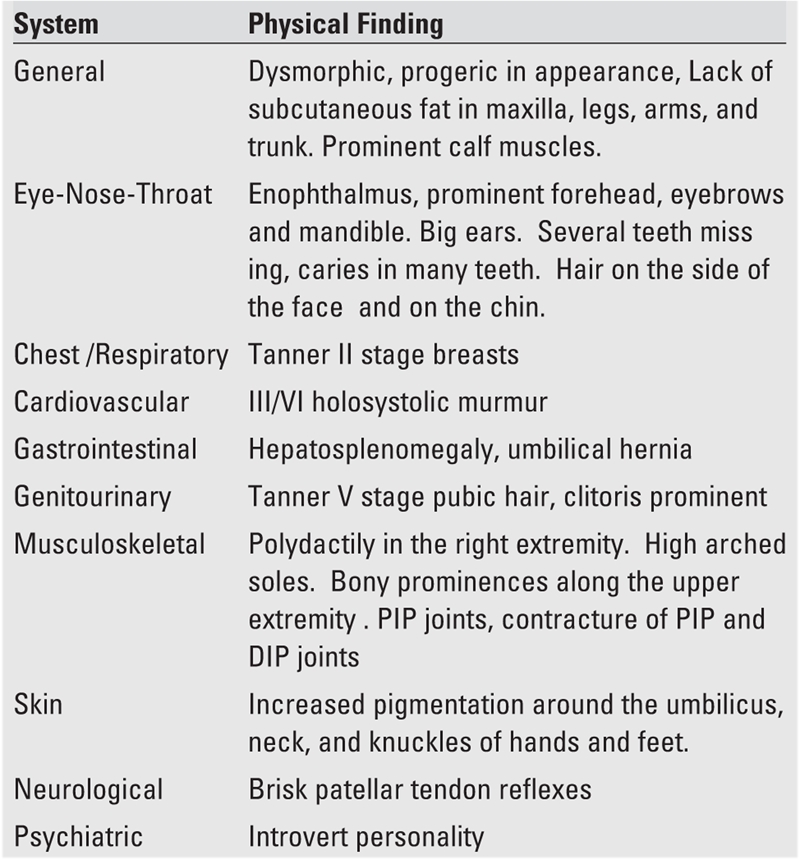
Physical findings of the patient

**Table 3:**
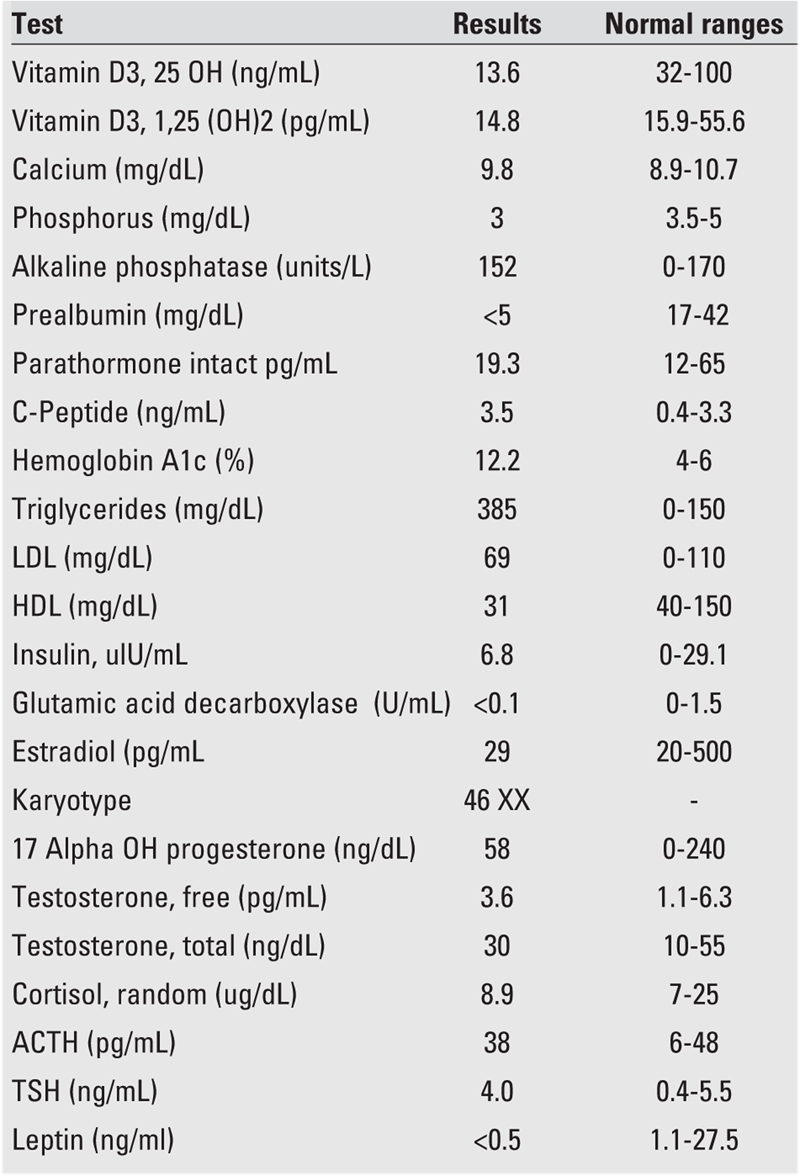
Laboratory data of the patient

## DISCUSSION

BSCL is a condition characterized by absence of functional adipocytes to store fat. As a result of this defect, fat is stored in tissues like muscle and liver. Hepatomegaly and muscle hypertrophy ensue due to excessive deposition of circulating triglycerides (5). Insulin resistance can become severe by the second decade of life, making the diabetes difficult to control with conventional therapy (3). Pathogenesis of the generalized lipodystrophy is secondary to adipocyte deficiency (6). The result is a distinct phenotypic appearance of generalized lipoatrophy that is exacerbated by the severity of the diabetes and subsequent insulin resistance. Major diagnostic criteria of CGL include trunk, limb, and facial lipoatrophy. Acromegaloid features are characteristic and consist of prognathism, enlarged hands/feet, macrogenitosomia, which are all thought to be a result of insulin cross−reacting with insulin−like growth factor−1 (IGF−1) receptors (5). Minor criteria include hypertrophic cardiomyopathy, psychomotor or mental retardation, hypertrichosis, hirsutism, and bone cysts with premature closure of the epiphyseal plates (3). Renal disorders may include nephromegaly and nephropathy (5). Our patient had most of the above features, except for bone cyst and cardiomyopathy. Her echocardiogram showed aortic regurgitation and left ventricular hypertrophy. Her left ventricular hypertrophy may be secondary to long−standing poorly controlled hypertension. Other features unique to our patient include polydactyly in the right upper and lower extremities as well as arthropathy with contractures at proximal interphalangeal (PIP) and distal interphalangeal (DIP) joints. The arthropathy may be secondary to poorly controlled long−standing diabetes or other undiagnosed etiology.

Infants with BSCL usually present with findings secondary to lipoatrophy, failure to thrive or gigantism, developmental delay, and dysmorphism (3). Alternate diagnoses to be considered in the differential diagnosis are progeria, Russell’s diencephalic syndrome, leprechaunism and lysosomal storage disorders (3).

Patients with CGL in the under−10 age group commonly present with accelerated growth, cognitive impairment, and abnormal fat distribution. By the second decade, symptoms of weight loss, polyuria, polydipsia, and polyphagia ensue and the diagnosis of diabetes is ultimately made. Our patient was diagnosed with diabetes at 8 years of age. Differential diagnosis in this age group is less extensive and includes: Dunningham lipodystrophy, a syndrome, which spares Bichat’s pads in the face; progeria, which is characterized by premature senility, sclerotic skin, joint contractures and alopecia; and Rabson−Medenhall syndrome, the prominent features of which are short stature, protuberant abdomen, abnormalities of teeth and nails, and pineal hyperplasia, with no organomegaly (3). Laboratory evaluation usually reflects insulin resistance, with impaired glucose tolerance, dyslipidemia and hyperinsulinemia. Our patient had normal fasting insulin levels, mildly elevated C−peptide, elevated triglyceride and low HDL cholesterol levels. Other features of insulin resistance in our patient were acanthosis nigricans and hirsutism. Hepatic steatosis will commonly result in a mild transaminitis as triglycerides deposit into the liver secondary to a paucity of generalized fat tissue (5). As a consequence of near−total loss of body fat, serum levels of adipocytokines such as leptin and adiponectin are low (15).

DNA testing can help differentiate subtypes of BSCL. Mutation of band 13 (called locus BSCL2) on the long arm of chromosome 11 prevents the coding of the enzyme seipin. This mutation is found in patients with congenital lipodystropy Type 2 (5). Conversely, a mutation on band 34 (called locus BSCL1) of the long arm of chromosome 9 inhibits the production of the enzyme 1−acylglycerol− 3−phosphate O−acyltransferase 2 (AGPAT2) (4, 5). This is indicative of congenital lipodystrophy Type 1. In contrast to type 1, unique features of type 2 are greater prevalence of cognitive dysfunction, cardiomyopathy, less lytic bone lesions and involvement of both metabolically active adipose tissue (found in most subcutaneous tissue sites, in intraabdominal, intrathoracic sites and in the bone marrow) and mechanical adipose tissue (located in the palms, soles of the exremities, under the scalp, in the retroorbital and periarticular regions) (5, 6). In Type 1, only metabolically active adipose tissue is involved. Our patient showed developmental delay, left ventricular hypertrophy and involvement of both metabolically active (less subcutaneous tissue, less intraabdominal fat revealed by abdominal CT scan) and mechanical adipose tissue (less fat in the palms and soles, enophthalmus). Management of lipodystrophy centers on controlling the diabetes, improving the insulin resistance, and reducing the triglyceride levels. Patients with CGL, as exemplified in this case report, are quite resistant to insulin therapy. Insulin doses as high as 1000 units daily may be required to control blood glucose levels (16) and even these doses may not suffice. Metformin has been shown to have some effect in helping patients reduce their appetite and improve symptoms of polycystic ovarian syndrome (PCOS) and hepatic steatosis (17). Our patient showed mild improvement in HbA1c on a combined regimen of insulin and metformin. Care should be taken in monitoring hepatic functions closely, as metformin is associated with hepatotoxicity. The most promising treatment for patients with CGL is recombinant leptin. This medication appears to improve insulin sensitivity, decrease triglyceride levels, and help control energy homeostasis. This results in less food intake, and lower fasting blood glucose levels as well as lower HbA1c levels (18). Some reports show normoglycemia on leptin therapy even after other discontinuing hypoglycemic agents (19).We are planning to enroll our patient in a leptin trial.

In summary, CGL is an uncommon genetic disorder. The patient described in this case report unfortunately went undiagnosed for many years. Maldistribution of body fat led to the pathological changes in the liver, kidneys, and muscles. Ultimately, diabetes and subsequent insulin resistance developed. With the exception of cardiomyopathy and lytic bone lesions, this patient meets all the major and minor criteria for the diagnosis of congenital generalized lipodystrophy type 2 (BSCL2).
